# Gonadotropin Activity during Early Folliculogenesis and Implications for Polycystic Ovarian Syndrome and Premature Ovarian Insufficiency: A Narrative Review

**DOI:** 10.3390/ijms25147520

**Published:** 2024-07-09

**Authors:** Salvatore Longobardi, Francesca Gioia Klinger, Wenjing Zheng, Maria Rosaria Campitiello, Thomas D’Hooghe, Antonio La Marca

**Affiliations:** 1 Global Clinical Development, Merck KGaA, 64293 Darmstadt, Germany; 2 Department of Histology and Embryology, University of Health Sciences, Saint Camillus International, Via di Sant’Alessandro 8, 00131 Rome, Italy; 3 Merck KGaA, 64293 Darmstadt, Germanythomas.dhooghe@merckgroup.com (T.D.); 4 Department of Obstetrics and Gynecology and Physiopathology of Human Reproduction, ASL Salerno, 84124 Salerno, Italy; 5 Department of Development and Regeneration, Biomedical Sciences Group, KU Leuven (University of Leuven), 3000 Leuven, Belgium; 6 Department of Maternal-Child and Adult Medical and Surgical Sciences, University of Modena and Reggio Emilia, 41125 Modena, Italy

**Keywords:** folliculogenesis, ovarian insufficiency, primordial follicle, antral stage, infertility, luteinizing hormone (LH), polycystic ovary syndrome (PCOS), premature ovarian insufficiency (POI)

## Abstract

Female fertility depends on the ovarian reserve of follicles, which is determined at birth. Primordial follicle development and oocyte maturation are regulated by multiple factors and pathways and classified into gonadotropin-independent and gonadotropin-dependent phases, according to the response to gonadotropins. Folliculogenesis has always been considered to be gonadotropin-dependent only from the antral stage, but evidence from the literature highlights the role of follicle-stimulating hormone (FSH) and luteinizing hormone (LH) during early folliculogenesis with a potential role in the progression of the pool of primordial follicles. Hormonal and molecular pathway alterations during the very earliest stages of folliculogenesis may be the root cause of anovulation in polycystic ovary syndrome (PCOS) and in PCOS-like phenotypes related to antiepileptic treatment. Excessive induction of primordial follicle activation can also lead to premature ovarian insufficiency (POI), a condition characterized by menopause in women before 40 years of age. Future treatments aiming to suppress initial recruitment or prevent the growth of resting follicles could help in prolonging female fertility, especially in women with PCOS or POI. This review will briefly introduce the impact of gonadotropins on early folliculogenesis. We will discuss the influence of LH on ovarian reserve and its potential role in PCOS and POI infertility.

## 1. Introduction

The mammalian ovarian reserve comprises a finite pool of primordial follicles representing the lifetime reproductive capacity of females. The total number of ovarian follicles is determined early in life, and the depletion of this pool leads to reproductive senescence [1]. Throughout a woman’s reproductive years, follicles are recruited and activated from the pool of dormant follicles. The fate of each follicle is controlled by endocrine as well as paracrine factors including stimulatory factors such as transforming growth factor beta (TGFβ) superfamily members, kit ligand (KITL) and leukemia inhibitory factor (LIF) as well as inhibitory factors such as cytokine stromal derived factor-1 and its receptor C-X-C chemokine receptor type 4 (CXCR4), the cell cycle regulator p2, anti-Müllerian hormone (AMH) and insulin [2,3]. Primordial follicles, consisting of an innermost oocyte surrounded by supportive somatic cells, are the reproductive units of the mammalian ovary that, once activated, enter an irreversible process of maturation including the primary, secondary and antral stages of development. In the antral stage, most follicles undergo atretic degeneration, whereas a few of them, under the cyclic gonadotropin stimulation that occurs after puberty, reach the preovulatory stage [1,4]. Interest in the early stages of folliculogenesis, the activation of primordial follicles and the further development throughout successive stages has increased in the past decade as it was recognized that the primordial pool is a resource that could potentially be used to increase reproductive efficiency and ameliorate infertility in women exposed to radiation or chemotherapies as well as those with poor prognosis and/or reduced ovarian reserve [5,6,7].

Polycystic ovary syndrome (PCOS) is a syndrome of ovarian dysfunction affecting 8–13% of reproductive-aged women [8]. The principal features of PCOS are oligoovulation and/or anovulation, hyperandrogenism and polycystic ovary morphology. Clinical manifestations may include irregular menstrual cycles, hirsutism, infertility, pregnancy complications and metabolic features (insulin resistance, metabolic syndrome, prediabetes, type 2 diabetes and cardiovascular risk factors) [8,9]. As PCOS is a syndrome rather than a disease, its diagnosis is made by the presence of two out of three of its main features (oligoovulation and/or anovulation, hyperandrogenism and polycystic ovary morphology) and the exclusion of other etiologies (congenital adrenal hyperplasia, androgen-secreting tumors, Cushing’s syndrome) [9]. A high percentage (55–75%) of women with PCOS have an elevated luteinizing hormone (LH)/follicle-stimulating hormone (FSH) ratio, presumably due to elevated levels of LH rather than reduced production of FSH [10]. In PCOS patients, variants of LH receptor (LHR) have been found; these LHR variants may diminish or enhance pituitary LH stimulation of ovarian theca and stroma cell T production, ovarian follicle development, LH-surge-induced ovulation and corpus luteum function, contributing to the genetic determination of PCOS phenotypes with specific reproductive pathophysiology [10].

Premature ovarian insufficiency (POI) is a clinical syndrome defined by loss of ovarian activity before the age of 40, with a prevalence of approximately 1% depending on population characteristics, such as ethnicity [11]. The causes of POI include chromosomal and genetic defects, autoimmune processes, chemotherapy, radiation, infections and surgery; however, many cases are idiopathic with unidentified causation [11]. POI is characterized by menstrual disturbance (amenorrhea or oligomenorrhea) with raised serum levels of gonadotropins FSH and LH and low serum estradiol levels [11]. Although proper diagnostic accuracy in POI is lacking, a combination of oligo/amenorrhea for ≥4 months, and an elevated FSH level > 25 international units (IU)/L on two occasions > 4 weeks apart in women < 40 years of age usually leads to POI diagnosis [11]. There is uncertainty over the role of FSH and LH in early folliculogenesis and in PCOS/POI-related infertility. Therefore, the aim of this narrative review is to analyze the current literature data on FSH and LH in early folliculogenesis and in the preservation of the primordial follicular pool as well as the potential relationship between FSH and LH perturbations and PCOS/POI.

## 2. A Brief Overview of the Early Human Female Folliculogenesis

Several efforts have been made over the past few decades to understand the endocrine, paracrine and autocrine factors acting in a spatial and temporal manner to regulate and coordinate the growth and development of the oocyte and its surrounding cells. Follicles develop through primordial, primary and secondary stages, also known as preantral follicles, before acquiring an antral cavity. In humans, the formation of primordial follicles starts at around 15–22 weeks of gestation and continues until just after birth, but only a small proportion of primordial follicles are recruited to the growing stages and contribute to female fertility during reproductive life. Other primordial follicles are likely to be under constant inhibitory influences of systemic and/or local origins to remain dormant, thus constituting a resting pool [1]. From 6–7 million oocytes in the ovaries in the fourth month of fetal development, only 1–2 million primordial follicles remain at birth due to apoptosis [12]. Follicle loss slowly continues after birth, so that at menarche about 300,000 to 400,000 follicles remain [12]. Through the reproductive years, approximately 1000 follicles are lost each month until the time of menopause, when the number of remaining follicles is <1000 [10]. The balance between the quiescence, death and activation of primordial follicles is essential in maintaining a proper reproductive lifespan [13,14].

Stimulatory and inhibitory signals for follicle activation are provided by autocrine factors from the follicle itself, paracrine factors from the surrounding granulosa cells, stromal cells and systemic factors [3]. It should be noted that much of this information comes from animal models. Stimulatory factors include members of the TGFβ superfamily, KITL, LIF, *FGF7*, bone morphogenetic protein 4 or 7 (BMP4 and BMP7), growth differentiation factor 9 (GDF9), androgens, insulin and interleukin-16 [3]. Inhibitory factors include cytokine stromal derived factor-1 and its receptor CXCR4, the cell cycle regulator p2, AMH, activin and inhibin. Patients with PCOS have elevated AMH levels, reflecting higher than usual numbers of preantral and small antral follicles and increased production by granulosa cells. This is likely to contribute to reduced follicle sensitivity to FSH and may result in follicular growth arrest [3].

**Figure 1:**
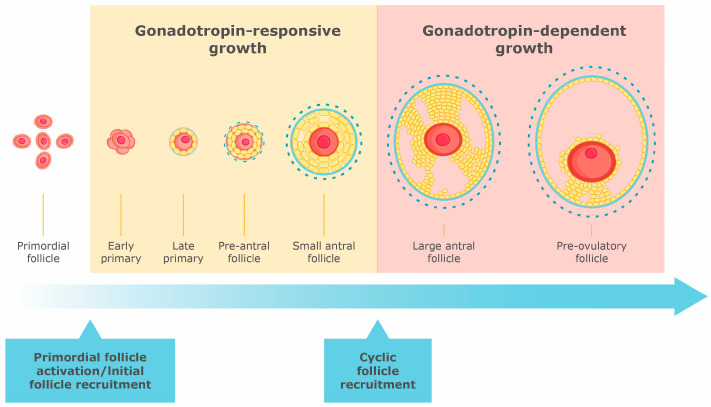
The role of gonadotropin throughout ovarian follicle development.

A primordial follicle is a structure enveloping a small primary oocyte (~25 μm) within a single layer of squamous granulosa cells on a basal lamina (Figure 1). The diameter of the human primordial follicle is approximately 30 μm [15]. When a primordial follicle enters the growth phase, its oocyte size increases, and together with granulosa cell proliferation and morphological changes, it becomes a primary follicle. The primary follicle is an oocyte surrounded by a single layer of cuboidal granulosa cells (Figure 1) [15], and primary follicles are found by 24 weeks of gestational age [1]. By 26 weeks of gestational age, some primary follicles progress to the secondary stage, and granulosa cells acquire the differential characteristics of epithelial cells [1]. Secondary follicles contain a fully grown oocyte surrounded by zona pellucida, five to eight layers of granulosa cells, a basal lamina and a theca interna and externa with associated blood vessels (Figure 1) [15].

The transition from a primary to a secondary follicle in the human ovary takes >120 days [1]. Antral follicles develop in the third trimester and are also seen postnatally when FSH levels are elevated [1]. At the antral stage, the follicle develops an “antrum”, a cavity containing follicular fluid, a plasma exudate that functions as a regulatory microenvironment of secretory products from both the oocyte and granulosa cells (Figure 1). Antral follicles grow to 1–10 mm in total diameter. The relative number and size of antral follicles vary between individuals and as a function of age and menstrual cycle stage [15]. The preantral to early antral transition is most susceptible to atresia [1], in which, during each menstrual cycle, cyclic gonadotropin stimulation causes a few antral follicles to reach the preovulatory Graafian stage (20 mm), thus releasing the mature oocyte for fertilization (Figure 1) [1,4]. It takes only 2 weeks for an antral follicle to become a dominant Graafian follicle [1].

During reproductive life, continuing growth of primordial and primary follicles into secondary and larger follicles leads to a gradual decrease in the original primordial follicle pool. In addition, the primordial follicle pool could also be decreased due to apoptosis of resting follicles [14]. More than 10 years before menopause, and concomitant with subtle increases in serum FSH and decreases in circulating inhibins, increasing percentages of follicles are lost from the resting pool. The diminishing follicle reserve serves as a ticking clock counting down the time to the onset of menopause. As the result of ovarian follicle exhaustion, menopause occurs at approximately 51 years of age, a time point that has been relatively constant for centuries [1].

Initial recruitment of primordial follicles to form primary follicles and the development of primary follicles into secondary follicles were classically thought to be regulated by gonadotropin-independent intraovarian factors [1], but accumulating data indicate that gonadotropins could also influence preantral follicular development [4,14]. The question of whether the extent and rate of early follicle growth are dependent on exposure to minute amounts of gonadotropins remains unsolved, and no robust evidence is available regarding when the human follicular cells start expressing gonadotropin FSH and LH receptors [16,17,18,19,20].

Human follicular development up to the antral stage continues throughout life until depletion of follicles around menopause, even under conditions in which endogenous gonadotropin release is diminished substantially, such as during the prepubertal childhood period or related to the use of steroid contraceptives [21,22]. In addition, follicle growth up to the early antral stage has been described in women with absent gonadotropin secretion, either due to hypophysectomy or to hypothalamic/pituitary failure [23]. Improved knowledge regarding the mechanisms regulating the initiation of primordial follicle growth, as well as atresia of early stages of follicle development, may shed more light on clinical conditions such as ovarian aging and premature ovarian failure, as well as the individual variability in menopausal age [13,17,24].

## 3. The Paracrine Control of Primordial Follicular Activation: Phosphoinositide 3-Kinase (PI3K)/Phosphatase and Tensin Homolog(PTEN) and Mammalian Target of Rapamycin Complex 1 (mTORC1)/KITL Pathways

**Figure 2:**
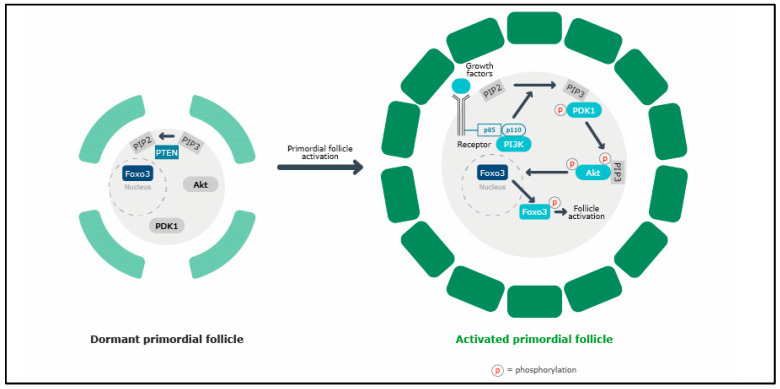
Primordial follicle activation driven by oocyte PI3K pathway. Growth factors, including KIT ligand, may activate the oocyte PI3K pathway and activate the dormant primordial follicles. Abolishing the dephosphorylation of PIP3 to PIP2 by oocyte-specific deletion of PTEN also triggers the activation of primordial follicles. On the other hand, suppressing the oocyte-specific PI3K-AKT-mTORC1 pathway inhibits the activation of primordial follicles. AKT, protein kinase B; FOXO3, forkhead box O3; mTORC1, mammalian target of rapamycin complex 1; PDK1, 3-phosphoinositide dependent protein kinase-1; PIP2; phosphatidylinositol (4,5)-bisphosphate; PIP3; phosphatidylinositol (3,4,5)-triphosphate; PI3K, phosphatidylinositol 3-kinase; PTEN, phosphatase and tensin homolog deleted on chromosome 10; p85, PI3K regulatory subunit; p110, PI3K catalytic subunit. Figure modified from Zhao Y, et al. *Cells* 2021, 10, 1491 [29].

While the later stages of folliculogenesis are well known to be gonadotropin-dependent, mostly under FSH control, early folliculogenesis is less well understood. Primordial follicle activation is a very dynamic and tightly controlled process, and despite the enormous progress that has been made, many molecular mechanisms are still not fully understood [25]. Primordial follicle activation is a result of the delicate interaction of pre-granulosa cells, oocytes and the follicular microenvironment. [2]. Knockout animal and in vitro cell culture studies have demonstrated that PI3K/PTEN signaling in oocytes governs primordial follicle activation (Figure 2) [25]. Different growth factors (e.g., KITL, insulin-like growth factor [IGF-1], epidermal growth factor [EGF], platelet-derived growth factor [PDGF] and vascular endothelial growth factor [VEGF]) stimulate the autophosphorylation of the intracellular regions of receptor tyrosine kinases (RTKs) expressed on the plasma membrane of the oocyte. Activated RTKs then stimulate the PI3K signaling pathway, leading to increases in phosphatidylinositol-3,4,5-triphosphate (PIP3) levels and serine/threonine protein kinase B (AKT) phosphorylation, which enhances cellular proliferation and survival. Activated AKT then migrates to the cell nucleus and suppresses FOXO3a, an apoptotic and cell-cycle-arrest transcriptional factor, promoting primordial follicle growth [25,26]. Treatment of ovaries with a cell-membrane-permeable peptide (740Y-P, designed based on the phosphorylated intracellular region of the PDGF receptor) stimulates AKT signaling and promotes the activation of primordial follicles [16]. PTEN is a negative regulator of the PI3K pathway. The *PTEN* gene encodes an enzyme that converts PIP3 to phosphatidylinositol 4,5-bisphosphate (PIP2), thus damping PI3K downstream signaling. Oocyte-specific deletion of the *PTEN* gene leads to global activation of primordial follicles, and treatment with a PTEN inhibitor, bpV(hopic), also promotes primordial follicle activation [27,28].

**Figure 3:**
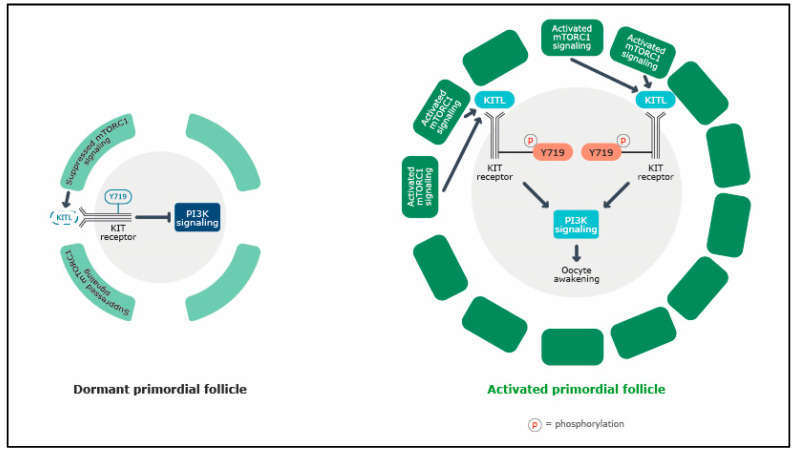
Primordial follicle activation driven by pre-granulosa cell mTORC1 pathway. In response to nutrients and factors, the mTORC1 pathway in the pre-granulosa cells of a primordial follicle is activated, leading to the synthesis of the KIT ligand. The KIT ligand binds to the membrane receptor of the oocytes of primordial follicles, activating the oocyte PI3K pathway and consequently the activation of the primordial follicle. KITL, KIT ligand;. Modified from Zhao Y, et al. *Cells* 2021, 10, 1491 [29].

Primordial follicle activation can also be regulated by the mTORC1 signaling pathway in primordial follicle granulosa cells (pfGCs; Figure 3). In response to stimulation by nutrients and other factors, mTORC1 signaling is activated in the pfGCs of selected primordial follicles, leading to the differentiation and proliferation of these cells. The activated mTORC1 signaling in pfGCs also stimulates the upregulation of the secretion of KITL, a receptor protein tyrosine kinase playing important roles in primordial follicle activation, oocyte growth and survival and granulosa cell proliferation in mammals [30]. KITL binds to KIT on the surface of the dormant oocyte, and this leads to the activation of intra-oocyte PI3K signaling. Activated PI3K signaling in oocytes awakens the dormant oocytes and stimulates their growth [13,30]. In mutant mice with oocyte-specific deletion of the tumor suppressor tuberous sclerosis complex 1 (TSC1), a negative regulator of mTORC1, the entire pool of primordial follicles is prematurely activated by mTORC1, leading to follicular depletion in early adulthood and causing POI [31]. In other genetically modified mouse models, ovarian PI3K signaling seems to be crucial for maintaining normal development and physiology of the ovary and for preventing its pathological conditions [32].

PI3K signaling in the oocyte determines the first wave of primordial follicle activation and contributes to female puberty. Concurrently, mTOR signaling in pre-granulosa cells determines female fertility throughout life [2]. mTOR and PI3K/PTEN/Akt are closely linked. As well as the activation of PI3K by mTOR-mediated KITL secretion mentioned above [13,30], they are also linked via the inhibition of tuberous sclerosis complex 2 (TSC2) by activated Akt. As TSC2 is a suppressor of mTOR, the inhibition of the mTOR pathway would be expected when PI3K is also inhibited [33]. An in vitro study with organotypic culture of cryopreserved whole murine ovaries treated with LY294002, a powerful PI3K inhibitor, or rapamycin, the specific mTOR inhibitor, showed that each drug acted on both the PI3K/PTEN/Akt and mTOR pathways and consequently inhibited the cryopreservation associated primordial follicle activation [34]. Moreover, an in vivo mouse study showed that the administration of rapamycin also inhibited the premature primordial follicle activation caused by the oocyte-specific deletion of the *PTEN* gene [35]. Together, these data confirm that the PI3K and mTOR pathways are closely linked during follicle activation.

## 4. Evidence for the Role of FSH and LH in the Development of Preantral Ovarian Follicles

In addition to local intraovarian factors and cytokines, particular attention has recently been paid to gonadotropins like FSH and LH, whose role in the development of preantral ovarian follicles remains controversial [1]. According to the old dogma, gonadotropins are required for antral follicle development, but not for the development of preantral follicles [24]. High FSH levels, usually occurring during the luteo-follicular transition, give rise to the continued growth of a limited number (cohort) of follicles. Subsequent development of this cohort during the follicular phase becomes dependent on continued stimulation by gonadotropins [24,36]. However, in vitro and in vivo studies indicate that the growth of preantral follicles could be enhanced by endogenous and exogenous gonadotropins. A reduction in gonadotropin levels in juvenile rats following either hypophysectomy or gonadotropin antagonist treatment leads to a decrease in ovarian weight, a reduced number of developing follicles, and an increase in atresia of remaining follicles compared to non-hypophysectomized rats [16,37]. In mature hypogonadal and gonadotropin-deficient mice expressing transgenic human FSH, transgenic FSH activity increases the overall development and/or survival of primordial follicles compared to both non-transgenic and wild-type mice, suggesting a role of FSH in the positive regulation of ovarian primordial follicle reserve [38]. In ovarian tissues from adult marmosets grafted freshly or following cryopreservation to ovariectomized nude mice, FSH treatment has been reported to prevent the depletion of primary follicles. Primary follicles with normal morphology were also increased in FSH-treated tissues in comparison with the control ones. In addition, FSH extended the cell metabolism and modulated mitogen-activated protein kinase (MAPK) signaling in the granulosa cells of preantral follicles, suggesting certain roles of FSH in early folliculogenesis up to the preantral stage [39].

Much less is known about the potential effects of LH on the growth of isolated preantral follicles. In mice, intact preantral small follicles (from 85 to 140 µm in diameter) require a slightly higher serum concentration of LH (optimal 10 mIU/mL) to grow and reach the antral stage in vitro than is needed once they reach the 150 µm threshold and beyond, when they can be cultured in a medium that contains FSH and 5% serum, which is virtually LH-free [40]. This finding confirms the critical role of LH for developmental progression through the preantral stage [40]. In a bovine co-culture model of granulosa and theca cells, it has been suggested that theca cell differentiation and early preantral follicle growth are dependent on subtle stimulation by LH [41]. Earlier studies in humans have demonstrated that FSH receptors are expressed in the granulosa cells of follicles from primary to later development stages and treatment with FSH and LH promotes preantral follicle growth [42,43,44,45].

Although acting on the same receptor, accumulating evidence suggests that in granulosa cells, human LH (hLH) binding has a greater impact on AKT and extracellular signal-regulated protein kinase (ERK1/2) phosphorylation than human chorionic gonadotropin (hCG) does [42]. Granulosa cells, together with oocyte and stromal cells, regulate primary follicular growth and development by a complex autocrine and paracrine relationship [15]. LH has higher activity than hCG during folliculogenesis, when the cell cycle regulators AKT and ERK play a crucial role, and these differences between hLH and hCG deserve consideration in the development of future therapeutic strategies [42].

A multi-center, randomized study explored the effects of pretreatment of small antral follicles with recombinant-human luteinizing hormone (r-hLH) in the absence of FSH, in women treated with a long protocol using a high-dose of a gonadotropin-releasing hormone agonist (GnRH-a). Results demonstrated increased development in the immediate term of small antral follicles in women receiving r-hLH treatment compared with the group without r-hLH treatment, showing a possible modest clinical benefit of the use of r-hLH in standard in vitro fertilization (IVF) [43]. In a clinical study including young patients (≤38 years old) with a poor ovarian response during at least two previous stimulation cycles, LH pretreatment produced a small increase in the number of oocytes retrieved (3.5 versus 2.4, *p* < 0.05) and a relevant improvement of their quality, assessed according to increased in vitro embryo development and implantation rate, suggesting that young poor responders may benefit from LH pretreatment before ovarian stimulation for ART [44].

Evidence from a case series of infertile women affected by hypothalamic amenorrhea (HA) who presented for fertility treatment with very low functional ovarian reserve shows that the administration of LH (at least 150–187.5 IU/day or every other day) may contribute to a clinically evident increase from baseline in both functional ovarian reserve (AFC) and AMH, and probably accounts for a positive effect of LH on the progression of small growing follicles through the antral stage [45]. Furthermore, there is emerging evidence that functional LH and FSH receptors are present in ovarian tissue before puberty in bovine species (FSH receptors) and in rodents (LH receptors). FSH receptors are expressed in somatic cells of bovine growing preantral follicles as early as the primary follicular stage of development, and preantral follicles respond to FSH by upregulating specific cellular functions and pathways (e.g., MAPK signaling) [46].

Quantitative changes in LHR mRNA levels were measured in the ovary during development in both normal and hypogonadal mice, which lack circulating gonadotropins [47]. Although shortened transcripts encoding the extracellular domain of the receptor were present from birth, the first full-length LHR was detected in rat and mouse ovarian somatic cells at 5 days post-partum and showed that the period of follicle development from the primary to mid/late secondary stages is associated with significant expression of LHR. [47]. The development of functional LHR in neonatal male and female rats is preceded by a change in the alternative splicing of LHR mRNA [48]. Using polymerase chain reaction multiplication of reverse-transcribed mRNA, the expression of the truncated LHR mRNA transcripts has been detected in the early stages of fetal rat gonads, providing evidence for the constitutive expression of one or more truncated messages of the *LHR* gene in undifferentiated gonadal cells [48]. Further research is therefore needed to investigate the role of gonadotropin or the gonadotropin receptor in early human folliculogenesis up until the antral stage. These studies may be useful in revealing unknown mechanisms behind follicular responses to LH related to developmental stages and provide new knowledge to be translated into clinical applications [43].

## 5. Gonadotropins and PCOS

In PCOS, increased LH and decreased FSH levels cause an increase in the LH/FSH ratio [49]. This leads to increased androgen secretion by ovarian theca cells, folliculogenesis arrest, accumulation of small antral follicles and higher AMH levels. AMH itself increases the LH/FSH ratio and impairs the conversion of androgens to estrogens, both of which result in increased androgen levels [49].

PCOS is a complication that women with epilepsy are susceptible to, with a prevalence of approximately 10–25% [50]. In women with epilepsy, focal epileptic discharges from the temporal lobe may have a direct influence on the function of the hypothalamic–pituitary axis [51]. Disturbance of central and/or peripheral control of hypothalamic–pituitary–gonadal axis and alteration of central neurotransmitters (gamma-aminobutyric acid [GABA], glutamate and serotonin) by epileptic discharges or antiepileptic drugs (AEDs), direct gonadal toxicity by AEDs and psychiatric/psychosocial factors are all causative factors for sexual, reproductive and gonadal abnormalities associated with epilepsy [52,53].

PCOS in epileptic women is commonly accompanied by hormonal alterations such as increases in serum free testosterone; dehydroepiandrosterone levels; free androgen index; free testosterone/LH ratio; sex hormone-binding globulin, estradiol, prolactin and LH; FSH levels; and LH/FSH ratio [50,54]. The connection between epilepsy and PCOS could be explained by the role of the brain in regulating the hypothalamic–pituitary–ovarian (HPO) axis, which controls the release of hormones, together with the neuroendocrine system [54]. Furthermore, valproic acid (VPA), primarily known for its use in the treatment of epilepsy, bipolar disorder and migraine headaches, may impact hormone levels, including LH, through its effects on the central nervous system by affecting neurotransmitter systems involved in the regulation of some hormone release. In this regard, VPA has been shown to affect GABA neurotransmission, which can influence hypothalamic–pituitary function, increasing LH serum levels. It was speculated that prolonged exposure to higher levels of LH may accelerate the rate of primordial/primary follicles entering folliculogenesis, promoting PCOS phenotypes among women with epilepsy treated with VPA. However, the exact mechanisms by which VPA may influence LH levels are not fully understood, and the available evidence is limited.

In an in vitro study on the impact of AEDs on ovarian follicular cells from prepubertal pigs, VPA, but not levetiracetam, caused a significant increase in LH-stimulated testosterone secretion and decreased FSH-stimulated estradiol secretion in gonadotropin-stimulated cultures compared to controls [55]. Another human in vitro study [55] evaluated the gene expression profiles of theca cells isolated from women with PCOS and controls without PCOS. The cells from the women without PCOS were treated or untreated with VPA in serum culture, while those from the women with PCOS were not treated with VPA. VPA-dependent alterations in gene expression were correlated to increased sensitivity of normal theca cells to insulin-dependent AKT/PKB phosphorylation, which has been shown to increase dehydroepiandrosterone and androstenedione synthesis in ovarian somatic cells. Increased AKT/PKB phosphorylation enhances somatic cell proliferation in the ovary and may provide a mechanism for theca cell hypertrophy, abnormal follicular development, and/or anovulation, which are characteristic phenotypes in women with PCOS [56]. In summary, studies indicate that VPA influences LH serum levels and that prolonged exposure to VPA elevates LH levels, which may stimulate the recruitment of primordial and primary follicles, accelerating the process of folliculogenesis. This can have implications for ovarian function and potentially contribute to the development of conditions like PCOS. In fact, studies have suggested a potential association between VPA use and an increased risk of PCOS development or exacerbation. This association may be mediated through VPA-induced alterations in LH levels and subsequent effects on ovarian function. By contrast, in adult rat ovaries, VPA administration significantly decreased the overall number of follicles, including non-ovulating large antral-like follicles and large cystic follicles, while a significant increase was observed in atretic follicles, and a nearly complete absence of corpora lutea was noted [57]. The levels of estradiol, progesterone, testosterone, FSH and LH decreased in VPA-treated rats compared to controls [54]. It is not clearly understood why VPA appears to decrease the number of antral follicles and serum LH levels in rats, whereas an increase in antral follicles and serum LH levels has been observed in humans, as explained in the section below [58].

The literature also suggests an association between VPA and the development of PCOS-like symptoms in women with epilepsy [59,60]. An epidemiological study evaluating polycystic ovary (PCO) and PCOS prevalence in epileptic women treated for 3.5 years with VPA, carbamazepine or phenobarbitone showed a significantly higher occurrence of PCOS in patients on VPA compared with normal population (15/40 [37.5%] patients in VPA group vs. 20/100 [20%] patients in normal population; *p* = 0.05) and patients on other AEDs (15/40 [37.5%] patients in VPA group vs. 18/100 [18%] patients in the other AEDs group; *p* = 0.02). These data highlight a potential association between VPA and reproductive system abnormalities in women [59]. VPA interferes at multiple levels within the endocrine system (including reducing the secretion of LH, FSH and prolactin and interference with peripheral endocrine hormones) [60]. Its administration as antiepileptic therapy, especially at pubertal age, leads to reproductive and sexual dysfunctions in both women and men [60].

Hepatic enzyme-inducing activity by AEDs, such as carbamazepine and phenytoin, may be most clearly linked to an increase in serum sex hormone-binding globulin in epileptic patients compared to matched controls [60]. A 5-year follow-up study in young women with epilepsy showed that treatment with VPA, an enzyme inhibitor, was associated with a 60% occurrence of PCOS [58,60], which is higher than the prevalence of approximately 37% reported over 3.5 years of follow-up discussed in the previous section [59]. In particular, women with epilepsy taking VPA, but not an oral contraceptive, were significantly more likely to have clinical biochemical evidence of PCOS, with raised LH and/or testosterone levels between days 2 and 6 of their menstrual cycle, than women who did not have epilepsy [58]. Further research is needed to clarify the effects of VPA on LH levels and its clinical implications in women. More comprehensive studies are required to determine the precise role of prolonged exposure to higher levels of LH in accelerating the rate of primordial/primary follicles entering folliculogenesis.

## 6. Gonadotropins and Premature Ovarian Insufficiency (POI)

In POI, increasing concentrations of gonadotrophins such as LH and FSH lead to premature luteinization of antral follicles and the downregulation of FSH receptors on granulosa cells [61]. Together, these processes limit the probability of normal ovulation [61]. POI resulting in female infertility is a frequent side effect of anticancer therapies, owing to the extreme sensitivity of the ovarian follicle reserve to the damaging effects of irradiation and chemotherapy on DNA [62,63]. Cyclophosphamide (CTX) has immunosuppressive effects and is used for the treatment of some cancers, such as breast and prostatic cancer, but significant drug toxicity has been noticed, particularly in the reproductive system [64,65]. CTX, although reported to promote the maturation of ovarian follicles [66], is mainly known to decrease follicular reserve by inducing apoptosis, ultimately leading to POI [67]. Moreover, in CTX–saline-treated mice, histological examinations showed that the number of normal follicles was significantly lower, the number of atretic follicles was significantly higher, the rate of apoptosis in ovarian granulosa cells was higher, serum E2 and progesterone levels were lower, and FSH and LH levels were higher compared with control mice (saline without CTX), altogether resulting in a phenotype similar to that of typical POI [64].

One strategy for gonadal function preservation is temporary ovarian suppression with gonadotropin-releasing hormone agonist (GnRHa) during chemotherapy. An in vitro study using immature cell–oocyte complexes (COCs) discarded from IVF cycles in healthy women showed that GnRHa was able to protect the oocytes from cyclophosphamide toxicity by inhibiting the apoptotic pathway [68]. Clinical studies from the literature provide evidence that GnRHa co-treatment reduces gonadotoxicity, with no serious side effects, becoming for many clinicians a clinically useful co-treatment for fertility preservation in women during chemotherapy [69]. A systematic review and meta-analysis was conducted to better assess the efficacy and safety of GnRHa co-treatment, measured by POI rate and post-treatment pregnancy rate, in patients with early breast cancer. A total of 873 patients from five trials were included in the analysis, in which premenopausal women with early breast cancer were randomly assigned to receive (neo)adjuvant chemotherapy alone or with concurrent GnRHa. Co-treatment with GnRHa reduced POI rates and increased the post-treatment pregnancy rates compared with chemotherapy alone (14.1% and 10.3% in the GnRHa group versus 30.9% and 5.5% in the chemotherapy-alone group, respectively) [70].

Intriguingly, LH treatment was recently suggested to indirectly protect prepubertal mouse follicles from the chemotoxic effects of cisplatin treatment [62]. In vitro and in vivo studies show that LH has the ability to protect the primordial follicle pool present in the ovaries of mice against cisplatin-induced apoptosis, preserving their future fertility in the reproductive age [71,72]. The actions of cisplatin and LH are clearly reciprocally influenced, suggesting that LH may modulate the composition of the extracellular environment in order to protect primordial/primary follicles from apoptosis [73]. In particular, in vitro LH treatment of prepubertal mouse ovarian fragments generates anti-apoptotic signals by a subset of ovarian somatic cells expressing LHR through cAMP/PKA and AKT pathways, reducing pro-apoptotic signals and accelerating the kinetics of cisplatin DNA repair occurring in cisplatin-treated oocytes [35,71,74]. Also, in mice in vivo, LH treatment has been shown to preserve the ovarian reserve and follicular development, avoid atresia, and restore ovulation and meiotic spindle configuration in mature oocytes of mice exposed to alkylating chemotherapy (cyclophosphamide and busulfan) at the primordial stage [72].

One therapeutic option for preserving follicle reserve in diseases such as POI and PCOS has emerged recently [75,76,77]. Ovarian tissue cryopreservation and transplantation (OTCT) enables ovarian function and fertility to be preserved in women and prepubertal girls who require chemotherapy and may also be useful in other conditions that impair female fertility [75,76,77]. In OTCT, samples of ovarian cortical tissue are obtained by laparoscopy or laparotomy and cryopreserved [76]. These samples contain thousands of follicles, and since ovarian stimulation is not needed, there are minimal delays in sampling, which can be important for patients requiring chemotherapy [77,78]. The transplantation of these ovarian samples back into patients has been shown to restore ovarian function in women and induce puberty in teenage girls with POI and has resulted in successful pregnancies [76,77].

## 7. Conclusions

The pool of oocytes in the mammalian ovary becomes fixed early in life; thus, ovarian senescence is linked to the dwindling supply and eventual exhaustion of the pool of primordial follicles [1]. The possibility of suppressing initial recruitment and preventing the growth of resting follicles could be the basis for designing treatments that would preserve the resting follicle pool, thus extending the female fertile period and delaying menopause [1]. On the other hand, both in vitro and in vivo studies have shown that the growth of preantral follicles can be enhanced by endogenous and exogenous gonadotropins, opening the way to treat patients with diminished ovarian reserve. Diminished ovarian reserve is an intermediate state where the ovarian reserve is not at its normal reproductive potential, and fertility is compromised [79], and could be defined as any of the risk factors for poor ovarian response (i.e., age over 40) and/or an abnormal ovarian reserve test (i.e., AFC < 5–7 follicles or AMH < 0.5–1.1 ng/mL) [80]. Taking this evidence together, we suggest that the development of follicles can be divided into gonadotropin-dependent, early gonadotropin-responsive and later gonadotropin-dependent stages [1,15], as shown in Figure 1. In addition, the elucidation of how and when gonadotropins participate in the initial recruitment of follicles could provide new scientific insights that could translate into novel medical treatments for women with diminished ovarian reserve, PCOS or POI.

## Data Availability

Not applicable.
